# The suitability of radiomics extracted from 4DCT motion‐compensated reconstruction

**DOI:** 10.1002/acm2.70254

**Published:** 2025-09-29

**Authors:** Chelsea A. H. Sargeant, Angela Davey, Alan McWilliam

**Affiliations:** ^1^ Division of Cancer Sciences, School of Medical Sciences, Faculty of Biology, Medicine and Health The University of Manchester Manchester UK; ^2^ Department of Radiotherapy Related Research The Christie NHS Foundation Trust Manchester UK

**Keywords:** 4DCT, lung cancer, motion compensation, radiomics, SBRT

## Abstract

**Purpose:**

The most appropriate 4DCT image for radiomics analysis remains uncertain, with options including individual respiratory phases and motion‐compensated (MC) reconstructions. This study compares radiomic features derived from MC reconstructions and patient‐specific, 4D phases, evaluating their influence on feature selection, robustness to registration algorithms and implications for radiomics analysis.

**Methods:**

This study included 223 NSCLC patients. MC reconstructions (MC_mean_ and MC_median_) were generated using three different registration algorithms and compared to a patient‐specific optimal 4D phase (TOp), defined as the phase where radiomic feature values exhibited the smallest variability. Ninety‐three features, extracted from the tumor region, underwent unsupervised selection to assess how image type influenced feature selection and redundancy. The impact of image type on distant failure prediction was evaluated using bootstrapped univariable Cox regression (*p* < 0.05) and multivariable modeling. Model performance was assessed across 500 bootstrap resamples, with feature selection frequency, concordance index (CI), and Akaike Information Criterion (AIC) recorded.

**Results:**

Over 60% of selected features differed between 4D phases and MC reconstructions, indicating image type influences feature selection. The proportion of remaining features varied: 11.8% (TOp), 15.1% (MC_mean_), and 12.9% (MC_median_). The single‐phase model, TOp, achieved a CI of 0.72 [0.64–0.77] and an AIC of 267.53, but did not demonstrate clear superiority over MC‐based models. Both MC_median_ and TOp showed modest improvements over the clinical model, suggesting both image types offer comparable prognostic potential. MC reconstructions were largely robust across registration algorithms, but MC_mean_ and MC_median_ reconstructions should not be used interchangeably.

**Conclusion:**

This study highlights differences in radiomic feature selection and predictive performance between MC reconstructions and individual 4DCT phases. MC reconstructions were a viable alternative, demonstrating robustness across registration algorithms. Both approaches can be integrated into radiomics pipelines, but image type selection should be carefully considered.

## 1 INTRODUCTION

For patients with early‐stage non‐small cell lung cancer (NSCLC), stereotactic ablative radiotherapy (SABR) is currently considered the standard of care in the treatment of inoperable patients, offering excellent tumor control, and reduced normal tissue toxicity.^1^, ^2^ Despite this, approximately 20% of patients will still experience distant metastasis within 5 years.^3^ Personalized treatments are thought to improve outcomes for NSCLC patients; if prognostic factors can be identified, a “one‐size‐fits‐all” treatment approach can be replaced by a refined plan tailored to the individual patient, based on tumor phenotype, and predicted therapeutic response.

Radiomics, the extraction and analysis of quantitative features from medical images, could play an important role in aiding prognosis.^4^ In recent years, radiomics has been extensively researched for patients with lung cancer, however, few studies consider the effects of tumor motion or four‐dimensional computed tomography (4D‐CT) data in their analysis. 4D‐CT is the standard of care for SABR planning since each phase in the respiratory cycle is captured and an accurate representation of a tumor at any point in time is provided. The inclusion of 4D‐CT in radiomics studies could mitigate the motion‐induced variability that the analysis of free‐breathing computed tomography (FB‐CT) data is limited by.^5^, ^6^ There is, however, uncertainty regarding how best to use 4D‐CT data; there are often ten respiratory phases available (T0, T10, … T90) as well as composite reconstructions, such as the average intensity projection (AIP), generated for radiotherapy planning. The breath‐hold (BH) technique could be considered the gold standard of motion management; however, success relies heavily on patient compliance and training. Both individual respiratory phases and motion‐compensated (MC) reconstructions are considered approximations of this technique.

The MC or mid‐position approach, developed by Wolthaus et al. has not been investigated for CT radiomics analysis or compared against other approaches.^7^, ^8^ The MC approach eliminates physical motion through the deformation of the internal structures in each phase to their time‐weighted mean position in the respiratory cycle. The frames are then averaged to create a single 3D‐CT scan.^7^ When generating MC scans, the 4D frames can be averaged using the mean or median over all phases. Both were investigated by Wolthaus et al. who concluded that although the median MC (MC_median_) results in sharper images and is less affected by artefacts, since outliers do not affect the median average, the tumor shape of the mean MC (MC_mean_) better resembles that of a BH CT scan. This reconstruction method minimises the motion‐induced artefacts and noise that are present in other 4D‐CT composites, resulting in sharper representations of the patient anatomy and a reduction in margins and toxicity as well as reduced interobserver variation when compared with the maximum intensity projection (MIP) technique.^9^ Clinically, the MC approach has been shown to maintain excellent local control rates following radiotherapy.^10^ However, this reconstruction approach may act to average the greyscale features within the tumor, reducing the potential for building predictive radiomics models compared to an individual phase.

The use of a single 4D phase for radiomics has also been suggested. The end‐of‐exhale phase (T50), effectively an under‐sampled approximation of the BH technique, has proven superior to both FB‐CT and AIP in feature analysis.^11^ The T50 phase is considered the most stable phase for most patients; however, a study by Davey et al. demonstrates that this is only true in around 35% of cases.^12^ Tailoring the phase selection to each patient successfully reduced the chance of performing radiomics analysis on a phase with an artefact. Additionally, in comparison to MC reconstructions, single‐phase images suffer from a reduced signal‐to‐noise ratio (SNR).^7^

In this study, we evaluated the suitability of using both mean and median motion compensation compared to a single 4D phase in radiomics analysis. Following a correlation‐led feature selection approach, we compared the downstream impact of implementations of motion compensation on radiomic models, as an exemplar we predict distant failure in early‐stage NSCLC. Since multiple deformable image registration (DIR) algorithms can be used, and limitations in registration accuracy can impact subtle image details,^9^ both MC_mean_ and MC_median_ reconstructions were generated with three DIR algorithms, Nifty, Elastix,^13^ and Galileo, to assess the robustness of feature extraction to the registration algorithm.

## 2 METHODS

### 2.1 Study population and follow‐up

From a single institution, 262 patients with early‐stage NSCLC treated with SABR to a single lung lesion during 2011–2017 were made available from a previous study.^12^ During clinical practice, a motion‐adapted gross tumor volume was contoured on the MIP for each patient by a radiation oncologist. The patients were treated with 54 Gy in 3 fractions or 60 Gy in 5 or 8 fractions.

Per UK guidelines,^14^ a clinical follow‐up was carried out 4–6 weeks post‐treatment. After this, patients were followed up quarterly for the first year, and bi‐annually thereafter. At follow‐up appointments, an FB‐CT was acquired, with an 18F‐FDG positron emission tomography (PET) scan and/or biopsy performed if recurrence was suspected. Distant failure was defined as recurrence in an uninvolved lobe, contralateral lung, or any other location in the body outside the lungs (metastasis). Time to distant failure was recorded from the start of treatment to the date of the first appointment showing recurrence. If there was no incidence of recurrence, patients were censored at their most recent follow‐up. Approval was granted to collect and analyse patient data (Research Ethics Committee references: 17/NW/0060 and 21/NW/0347); follow‐up data was collected retrospectively by the clinical team.

Available clinical variables were age, sex, T‐stage, tumor lobe location, ECOG performance status (scored from 0 to 5 to assess functional status), Adult Comorbidity Evaluation‐27 (ACE‐27) score (marked mild‐severe based on presence and severity of pre‐existing medical conditions), and histological subtype.

Patients with tumor volumes below 2 cm^3^ were excluded from the analysis to improve the reliability of radiomic feature extraction. Small tumors are prone to partial volume effects, where voxel intensities are distorted by the inclusion of multiple tissue types, and segmentation inaccuracies, both of which degrade the stability and reproducibility of radiomic features. These challenges are well‐documented in the radiomics literature^15^, ^16^ and several studies have used minimum volume thresholds to address them. While some use more conservative thresholds (e.g., 10 cm^3^), we opted for a lower cut‐off due to disease type (early‐stage NSCLC) and treatment (SABR), which commonly involve smaller lesions. The 2 cm^3^ threshold provided a balance between excluding technically unreliable cases and retaining a sufficient cohort for analysis. Patients with missing clinical data were excluded from the analysis with outcome.

Thirty‐nine patients were excluded before analysis: one had missing image/planning information, one had synchronous tumors at treatment, and the other 37 had tumor volume $<2\;cm^{3}$. Median follow‐up time for the remaining 223 patients was 14 months (95% CI 12–15 months), and 38 patients experienced distant failure.

The 223 patients recorded in Table 1 were included in the comparison and unsupervised feature selection stage of this study; however, 176 with complete, pre‐selected, clinical information were included in the final supervised feature selection and model‐building analysis. Of these 176 patients, 32 (18%) experienced distant failure. Comorbidity score and histological subtype were not included in the multivariable analysis. In the analysis of lobe location, “middle” and “upper” were combined for ease of comparison and “ECOG 0” was combined with “ECOG 1” since only two patients were in the lowest performance status group.

**TABLE 1:** Patient demographics for the 223 patients used for initial analysis.

Variable	Category	Count	Total
Age	Median (Range)	76 (45–93)	(100%)
Sex	Male Female	120 103	223 (100%)
T‐Stage	T1 T2 T3	124 76 1	201 (90.1%)
Tumor volume (cm^3)	Median (Range)	6.67 (2.07–41.92)	223 (100%)
Tumor lobe location	Lower Upper	73 144	217 (97.3%)
Histological subtype	Adenocarcinoma, NOS Squamous cell carcinoma, NOS, Carcinoma, NOS Other	43 40 16 8	107 (48%)
Tumor motion amplitude (mm)	Median (Range)	5.81 (0‐34.3)	223 (100%)
Performance status (ECOG)	0 1 2 3	2 65 106 21	194 (87%)
Comorbidity score (ACE‐27)	Severe Moderate Mild None	62 62 36 6	166 (74.4%)

Some records were incomplete, as a result “Total” column records the number (and percentage) of patients with complete data for each variable.

Abbreviations: ACE‐27, Adult Comorbidity Evaluation 27; ECOG, Eastern Cooperative Oncology Group.

### 2.2 Image acquisition and reconstruction

For each patient, routine pre‐treatment 4D‐CT scans were acquired, and the respiratory signal was measured using Phillips Brilliance‐CT Big Bore Oncology and Phillips Bellows Device, following standard imaging protocol at The Christie NHS Foundation Trust (120 kV and 800 mAs). All scans were reconstructed to a 512 × 512 image with a slice thickness of 3 mm, most images had a pixel size of 1.17 mm (range: 0.98–1.37 mm).

The 4D data was organized to create a full CT scan at ten evenly spaced time points during the respiratory cycle: T0, T10, …T90. T50 is often accepted as the phase representing end‐of‐exhale; however, this phase does not consistently correspond to the same respiratory state across all patients due to variations in breathing patterns and phase‐binning techniques. To account for this variability, a personalized phase selection approach was used to determine the most stable or “optimal” phase (T_Op_) for each patient, as described in a previous study.^12^

The optimal phase was defined as the phase where radiomic feature values exhibited the smallest sum of differences when compared to its two adjacent phases. This method accounts for individual variations in respiratory motion and minimises the impact of phase‐related artefacts. The stability of features was evaluated across 93 first‐order and texture features extracted from both an original and filtered image, which used a Laplacian of Gaussian (LoG) filter with sigma, σ, as 1.5 mm. LoG‐filtered features were included only during this phase selection step to enhance sensitivity to motion‐induced artefacts and provide a robust basis for identifying the most stable phase. All subsequent radiomic analyses and model development steps used only features extracted from the original (unfiltered) images. The features were extracted from both the tumor and the surrounding tissue.^12^

To generate the motion‐compensated scans, an approach described by McWilliam et al.^17^ was modified. DIR was applied to align each phase to a consistent reference phase, reducing motion‐related variability and allowing for the generation of motion‐compensated mean and median datasets. The process involved the following steps:

- Each phase of the 4DCT was deformably registered to the T50 phase, which served as the reference phase.
- The DICOM deformation vector field (DVF) was exported to a conquest DICOM server, and the mean DVF was calculated.
- The calculated mean DVF was then subtracted from each DVF, and the modified DVFs were then applied to deform the corresponding datasets to the mid‐position.
- The deformed phase datasets were subsequently averaged to create the MC_mean_ and MC_median_ datasets, respectively.

DIR was performed using tools from the NiftyReg deformable registration package.^18^ To further investigate whether differences in deformable registration package impact radiomic feature selection and model building, MC_mean_ and MC_median_ scans were also generated using the Elastix package^13^ and Galileo, an in‐house implementation of the DIR method described by Wolthaus et al.^8^ There is some uncertainty associated with DIR; however, for this task, it is likely to be very small since the registration is intra‐patient, between the 4D phases. However, the registration quality was visually assessed to identify cases of gross errors, with these corrected or removed as required.

**FIGURE 1:**
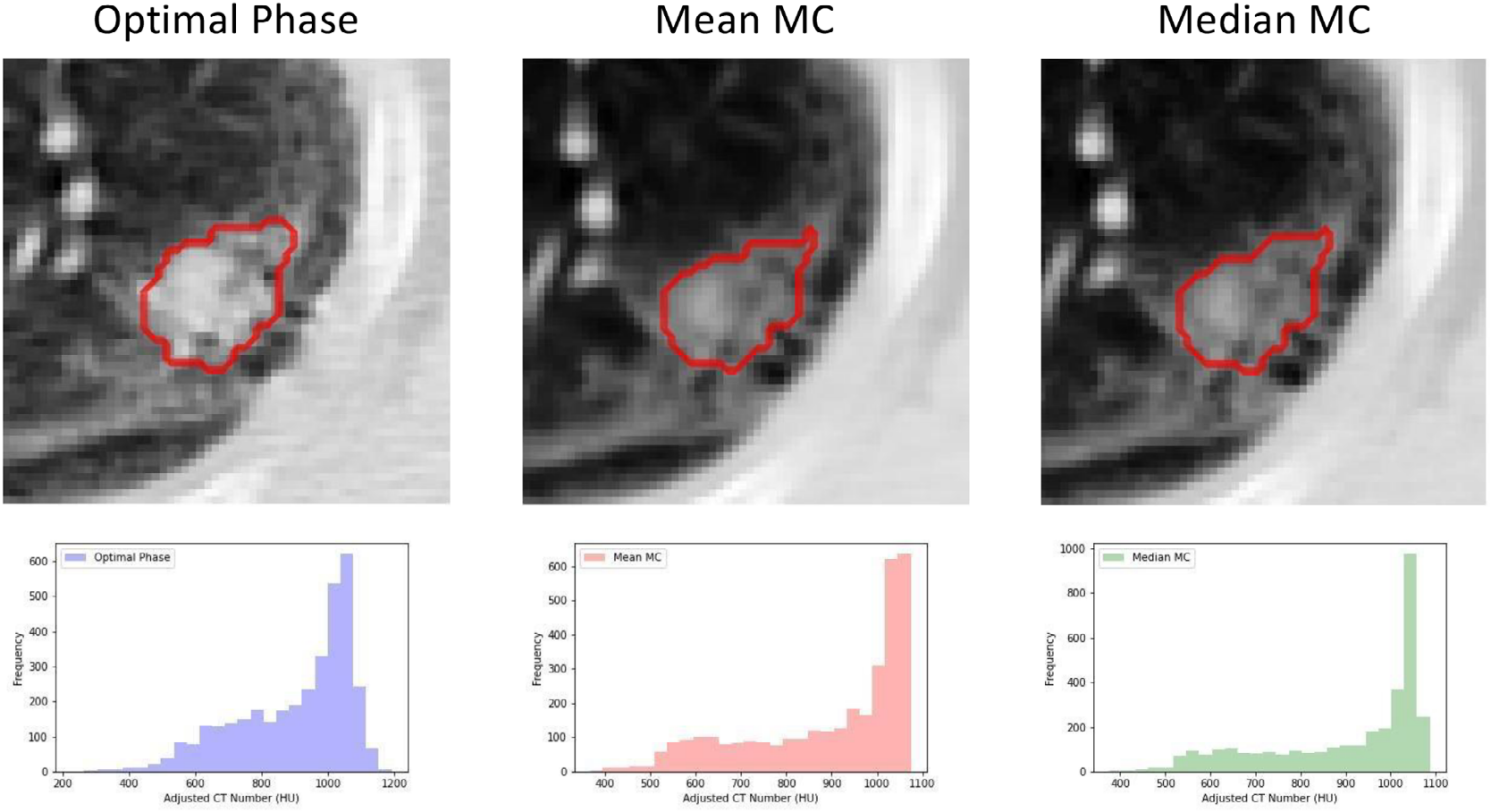
The region of interest (ROI) within which radiomics features were extracted are outlined for each approach; optimal phase (T50 in this case), and the MC_mean_ and MC_median_ reconstructions (top) with histogram of pixel values within the ROI (bottom). Attention should be drawn to the variability between image type, the optimal phase appears noisier than the MC reconstructions. Also, the slight differences in contour which are a result of the thresholding procedure.

Figure 1 shows an example slice of each image type; optimal phase, MC_mean_ and MC_median_, for a single patient. In this example, the optimal phase selected was the exhale phase (T50). On each image, the ROIs within which the radiomic features were extracted are displayed. Also shown are histograms of HU values within ROI to demonstrate variability between image types.

The optimal phase for each patient was determined using a data‐driven approach developed by Davey et al.^12^ For most patients, the phases close to exhale were the most stable phase, the frequency of selected phases is displayed in S1 Section 1, Figure 1.

### 2.3 Target volume segmentation

For each patient, a motion‐adapted gross target volume was available, which captures the range of motion of the tumor over the breathing cycle and is delineated clinically on the MIP scan. For each patient, we retrospectively generated the gross tumor volume (GTV) on a reference phase (T50) from the motion adapted GTV using an in‐house validated method.^19^ Briefly, the motion adapted GTV was rigidly translated by the rigid registration translation matrix between each phase and the reference phase (50%). The union of motion adapted GTV translated to each phase was then defined as the GTV. Local rigid registration was then performed to map the tumor position on each phase and the new GTV was resampled as a mask on the selected optimal phase (T_Op_) and the MC reconstructions for each patient.

Before feature extraction, the GTVs were refined by a thresholding procedure to define the GTV mask for each image type. A lower threshold of ‐500 Hounsfield Units (HU) was used to minimize the amount of lung tissue captured by the mask. Following this, morphological dilation and erosion were performed in Python (version 3.6.9) to remove cavities in the masks. Thresholding can influence radiomic feature values by altering the size and boundary sharpness of the segmented region, particularly affecting shape‐ and edge‐based features. To minimise segmentation‐induced variability in radiomic features, the same thresholding and morphological processing steps were applied consistently across all image types and patients. While a formal sensitivity analysis was not performed, this standardized approach was intended to reduce inter‐case variability introduced by segmentation differences. The mask was used solely to define the region of interest (ROI) from which to extract features.

### 2.4 Feature extraction

A set of first‐order and texture radiomic features were extracted from the ROI in each image type; T_Op_, MC_mean_, and MC_median_, using PyRadiomics (version 2.2.0), an open‐source software used in Python (version 3.6.9).^20^ Most features in this software are compliant with the Image Biomarker Standardisation Initiative (IBSI).^21^ The chosen features, extracted with default settings, were: first order, symmetrical grey level co‐occurrence matrix (GLCM), grey level run length matrix (GLRLM), grey level size zone matrix (GLSZM), neighboring grey tone difference matrix (NGTDM), and grey level dependence matrix (GLDM). No image filters were applied, resulting in a total of 93 features per approach. Since all patient images are of the same slice thickness (3 mm), the feature extraction was performed on the original voxel spacing. Texture feature extraction was performed using a fixed bin size approach with a bin size of 25HU, as recommended by the IBSI.^21^ The impact of variations in bin size and image preprocessing, while not the focus of this study, is acknowledged as a potential source of variability in radiomic features.

### 2.5 Optimal phase versus motion‐compensated reconstruction

To directly compare the differences in features extracted from each approach, a one‐way analysis of variance (ANOVA) test was conducted. A *p*‐value of 0.05 was considered significant. All statistical analysis was performed in R version 4.0.2.

#### 2.5.1 Unsupervised feature selection

Feature values for each image type were standardised to mean zero and unit variance before being subjected to an unsupervised selection process to reduce the risk of introducing redundant information. Correlations between features and number of voxels (representative of tumor volume) were investigated with Spearman rank correlation coefficient (ρ); those with ρ≥0.6 were excluded. This cut‐off preserves features with low‐to‐moderate correlation with volume while removing those strongly influenced by tumor size.

Correlations between the surviving features were then investigated for each approach, again with the Spearman rank correlation coefficient. Based on existing literature,^22^ a threshold of ρ≥0.8 was chosen to indicate strong correlation in correlated feature pairs and the feature in the pair with the largest average absolute correlation coefficient was removed to prevent redundancy.

#### 2.5.2 Supervised feature selection/evaluation of clinical and radiomic models for distant failure prediction

Remaining features were assessed for their ability to predict distant failure using a two‐step bootstrapping approach, designed to minimize overfitting and enhance model robustness:

1. Bootstrapped feature selection: A univariable Cox proportional hazards analysis was performed using 500 bootstrap iterations, where features were repeatedly resampled. Features that demonstrated a consistent statistically significant association (*p* < 0.05) with distant failure across resamples were recorded. The most frequently selected features were retained for multivariable modeling, with the final feature sets determined by the median signature size across bootstrap iterations.
2. Bootstrapped internal validation of model performance: The selected radiomic features were included in a multivariable Cox model alongside pre‐selected clinical variables (tumor volume, tumor motion, lobe location, performance status, T‐stage, sex, and age). Model performance was assessed using:
3. Concordance index (CI) estimated over 500 bootstrap iterations.
4. Akaike Information Criterion (AIC)
5. Hazard ratios with 95% confidence intervals.

This approach serves as an internal validation approach, ensuring that feature selection and model evaluation are conducted independently.

### 2.6 Dependency on DIR algorithm

Finally, the above analysis (unsupervised and supervised feature selection, model assessment) was also conducted for MC_mean_ and MC_median_ scans generated using both Elastix and Galileo registration algorithms to ensure feature selection is robust to variation in the MC generation process. Registration parameters were independently optimised for each DIR algorithm based on their respective default settings and documentation. In addition to the steps above, comparisons were made between each MC registration method to test for differences throughout the process.

## 3 RESULTS

### 3.1 Feature extraction

Features were extracted from the 3D ROI for each image type. The thresholding step described in Section 2C resulted in slight changes in volume for each approach. For this reason, the number of voxels within the mask for each image type was used as an alternative to tumor volume going forward. The average absolute change in volume, in number of voxels, for each image type can be found in S1 Section 2, Table 1.

The initial investigation, where the 93 features extracted from each approach were compared, indicated that most features (∼ 61%) differ between the individual phase and the MC reconstructions. The quantity and percentage of features that differed significantly between the compared approaches are displayed in Table 2.

**TABLE 2:** The number and percentage of features that are significantly different when testing feature values between the optimal phase and MC reconstructions using an ANOVA test.

Feature class	Optimal vs. MC_mean_	Optimal vs. MC_median_	MC_mean_ vs. MC_median_
First Order	12 (66.7%)	10 (61.1%)	0 (0%)
GLCM	17 (70.8%)	17 (70.8%)	0 (0%)
GLRLM	10 (62.5%)	10 (62.5%)	0 (0%)
GLDM	9 (64.3%)	9 (64.3%)	0 (0%)
GLSZM	10 (62.5%)	9 (56.3%)	2 (12.5%)
NGTDM	4 (80%)	4 (80%)	0 (0%)
Total	62 (66.7%)	59 (63.4%)	2 (2.2%)

In contrast to this, only a small number (2%) differs between the MC_mean_ and MC_median_ reconstruction. Similar results for the Elastix and Galileo MC reconstructions are found in S1 Section 3.

**FIGURE 2:**
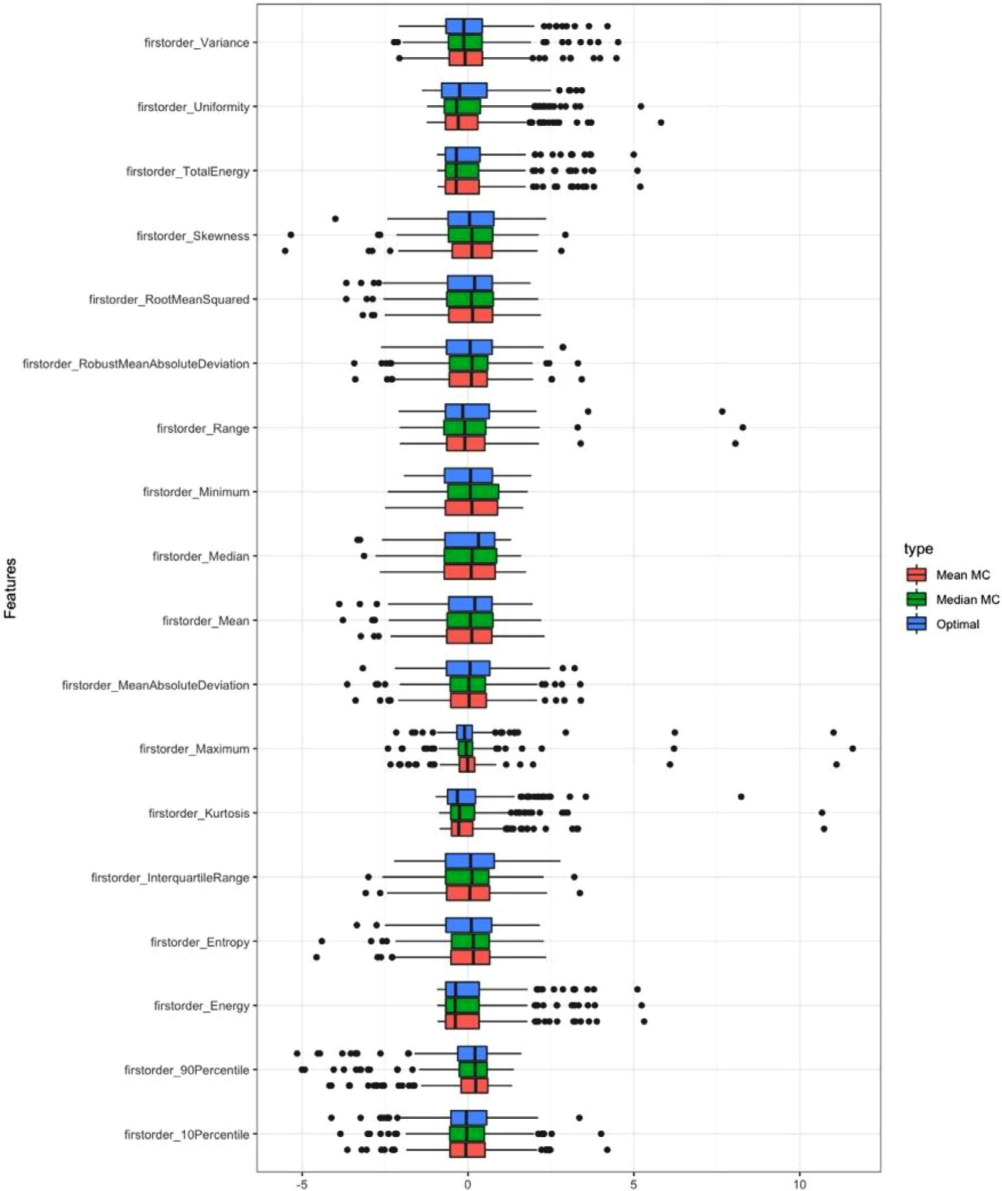
A comparison of first order feature values extracted from the optimal phase and the MC_mean_ and MC_median_ reconstructions using NiftyReg.

**FIGURE 3:**
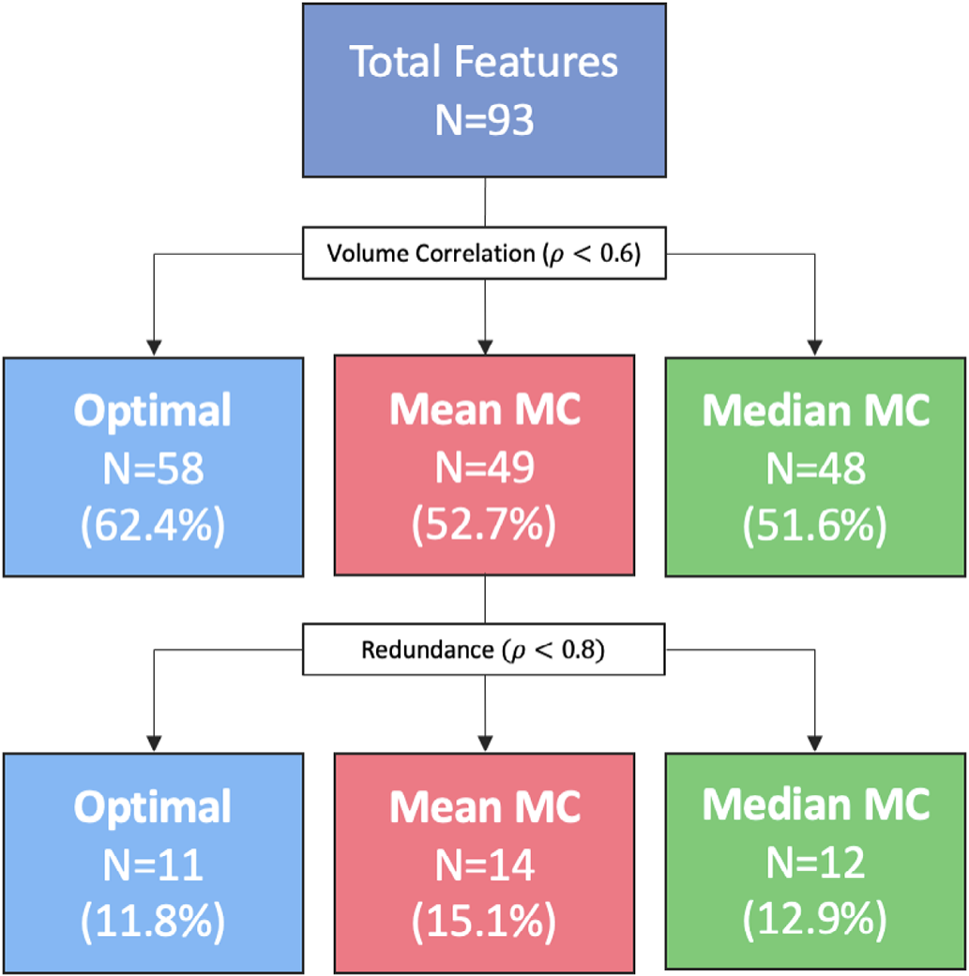
The number of features (*N*) surviving after each feature selection stage for all three image types.

**FIGURE 4:**
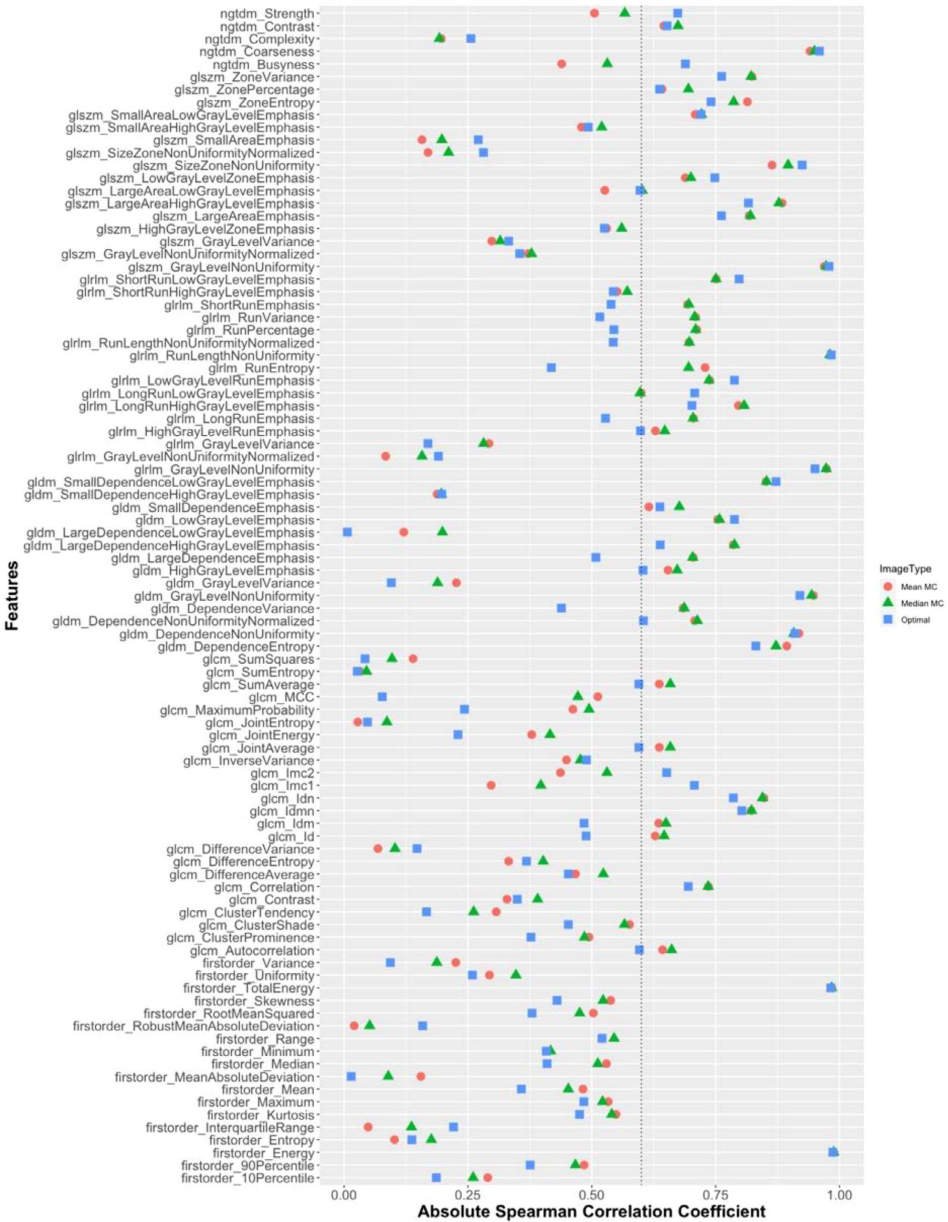
Absolute Spearman rank correlation coefficient for each feature with tumor volume. The dotted line represents the cut‐off threshold of ρ ≥ 0.6. Features with coefficients above this (to the right of the dotted line) are considered to have a high correlation with tumor volume and are excluded. Features with coefficients below the cut‐off are considered to have a weaker correlation with tumor volume and remain in the analysis.

**FIGURE 5:**
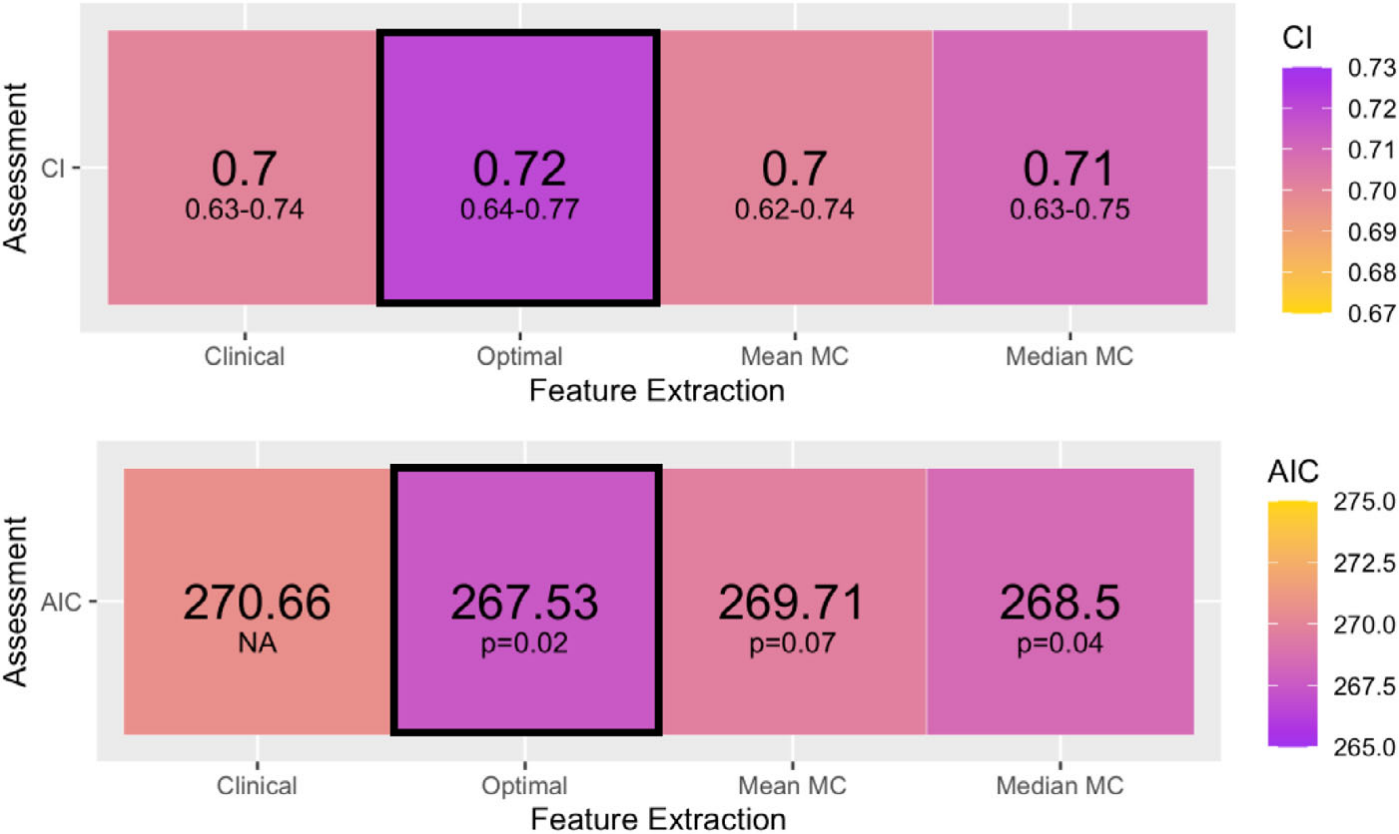
Median and 95% confidence interval of the concordance index (CI) across 500 bootstrap resamples (top), and the Akaike Information Criterion (AIC) for each model (bottom). In both, the best‐performing model is outlined by a black box.

**FIGURE 6:**
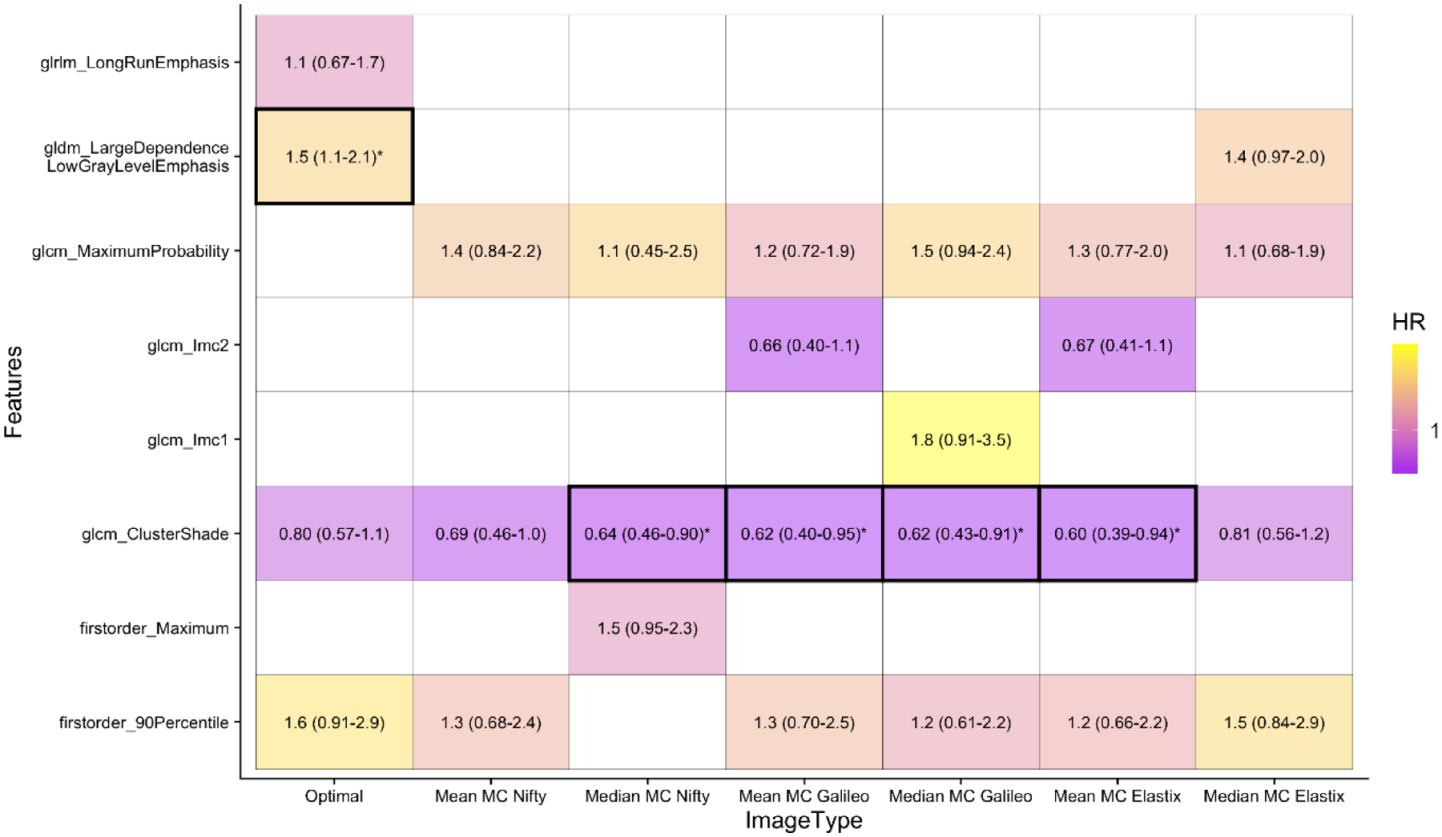
Hazard ratios and 95% confidence intervals from multivariable analysis for features and distant failure. A *p*‐value of 0.05 is considered significant, and significance is indicated by an asterisk and outlined box. Also included are results for Galileo and Elastix MC models.

The features that varied between the two MC scans were *GLSZM Size Zone Non Uniformity Normalised* (*p* = 0.02) and *GLSZM Small Area Emphasis* (*p* = 0.02). These features are significantly different in all comparisons. The differences in the first order feature values are visualized in Figure 2; it is apparent that even “simple” metrics can differ depending on the image from which they are extracted. Similar figures for all other feature types can be found in S1 Section 3, Figures 3, 4, 5, 6.

### 3.2 Feature selection

The number and percentage of features remaining at each stage of unsupervised feature selection is depicted in Figure 3.

Following correlation with volume, more features were removed for the MC reconstructions, with 62.4% remaining for T_Op_ and an average of 52.2% for MC. The absolute Spearman rank correlation coefficient for each feature is displayed in Figure 4, highlighting the differences in correlation for each image type. The type of 4D‐CT image used for radiomics alters the number of features with a strong‐to‐moderate dependence on volume. Of the remaining “volume‐independent” features, 45 were common across all approaches.

Following an assessment of inter‐feature correlations, 11.8% of features remained for T_Op_ and 15.1% for both MC_mean_ and MC_median_. For the MC reconstructions, more features were correlated with volume than in the optimal phase, but less were correlated with each other. Overall, fewer features were eliminated for the MC reconstructions.

There is also variability in the features that remain, just six features were common across all three image reconstructions: First Order Kurtosis, GLCM Cluster Shade, GLCM Difference Variance, GLCM Inverse Variance, GLCM Maximal Correlation Coefficient (MCC), GLDM Large Dependence Low Gray Level Emphasis.

Following supervised feature selection, the median signature size ranged from 3 to 4 features across image type. The median size was used to select the top‐ranked features following bootstrapping.

### 3.3 Dependency on DIR algorithm

There are no significant differences in features values between like scans in a paired comparison of each non‐rigid algorithm tested: Nifty, Elastix, and Galileo, suggesting that radiomic feature values are robust to changes in reconstruction algorithm.

Features selected from the MC_median_ are most sensitive to reconstruction algorithm, with less consistency in the number of features remaining after unsupervised feature selection (S1 Section 4, Figure 7). Also, fewer features survive unsupervised selection compared to MC_mean_, however, in all cases more survive for MC images than the single optimal phase. About 78.6% of MC_mean_ features remain for all registration algorithms compared to an average of 72.1% for MC_median_ features.

### 3.4 Evaluation of clinical and radiomic models for distant failure prediction

The CI and AIC for the clinical‐radiomics models, internally validated using bootstrapped resampling, are displayed in Figure 5. The baseline clinical model (reported in S1 Section 5 , Table 3) identified tumor volume, tumor motion, biological sex, tumor lobe location, and T‐stage as significant predictors of distant failure. On average, all radiomic models outperform the clinical model. The optimal phase and MC_median_ demonstrated small but statistically significant improvements in AIC compared to the clinical model (p=0.02 and p=0.04, respectively).

The optimal single‐phase model was the best‐fitting model, achieving the lowest AIC (267.53) and highest median CI (0.72, 95% CI: 0.64–0.77). Relative to this, the MC_median_ model yielded a ΔAIC of 0.97, indicating a comparable fit. The MC_mean_ model had a ΔAIC of 2.18, suggesting slightly reduced support, while the clinical model had a ΔAIC of 3.13, indicating a comparatively poorer fit to the data. These values align with conventional AIC interpretation thresholds, where ΔAIC < 2 indicates models that are strongly competing for best fit.

To formally assess differences in discriminatory performance, we applied a statistical test of CI difference across the models. This confirmed that each radiomic model significantly outperformed the clinical model (p<0.001), while comparisons between radiomic models were not statistically significant (p>0.34). Taken together, these findings suggest that while radiomic models improve upon the clinical baseline, they demonstrate comparable predictive performance with no single model demonstrating definitive superiority.

This trend remained consistent for the Elastix MC models. Although these models demonstrated improved performance within the available dataset, further external validation is required to determine their true prognostic utility.

Displayed in Figure 6 are the multivariable hazard ratio and significance values for features included in each of the models. Only one feature is selected in all models: *GLCM Cluster Shade*. This feature is a measure of skewness where an increased value implies greater asymmetry about the mean. For MC images, a significantly reduced risk of distant failure following SABR is indicated, though this observation requires further validation. This feature is robust to image type, but the feature value changes significantly between MC approaches according to the ANOVA test. No features selected for the MC_mean_ NiftyReg or MC_median_ Elastix model were significantly prognostic of distant failure. The use of the NiftyReg algorithm resulted in a reduced signature size compared to all other models, however, each MC model offered similar prognostic performance. CI and AIC performance can be found in S1 Section 4, Figure 5 for Galileo MC and Elastix MC models.

## 4 DISCUSSION

### 4.1 Methodological insights

In this study, we investigated the potential of using motion‐reconstructed 4D‐CT for radiomics feature extraction in NSCLC patients. MC reconstructions were compared directly with a patient‐tailored single 4D‐CT phase to determine the impact of motion compensation and its downstream impact on radiomics analysis. Significant differences were observed in radiomic feature values and their correlations with volume and other features across reconstruction methods, indicating that while MC reconstructions can be used within radiomic analysis, they should not be used in combination with other reconstructions. Similarly, models developed with one reconstruction may not generalise to other reconstructions. We applied commonly used unsupervised feature selection techniques, accounting for volume dependence and feature redundancy, as recommended by the ISBI.^21^ Although emerging approaches such as minimum redundancy, maximum relevancy (MRMR) and LASSO can effectively select features with high similarity, no consensus exists on the best feature selection method for radiomics and benchmarking studies find no consistent benefit to using one over another.^23^ Further testing of these techniques as they become a part of established methodologies may be of interest. However, similar results are expected between reconstructions, as each method fundamentally compares similarities between features.

Tools like PyRadiomics allow for the extraction, of over 1000 radiomic features using various image filters. In this study, filters were deliberately excluded to maintain a clear focus on comparing MC reconstructions with individual 4DCT phases. While the influence of additional filters warrants further investigation, this study concentrated on how reconstruction methods (MC_mean_, MC_median_, and T_Op_) influence radiomic feature extraction. Shape features were also excluded, as motion‐adapted tumor contours, representing the full motion range, produce smoother boundaries and reduce the reliability of shape‐based metrics. Instead, we focused on intensity‐ and texture‐based features. Texture features are sensitive to factors such as image quality, bin size, and processing variations, which can affect their reproducibility and generalisability. While these sensitivities are well‐documented, we emphasize the need for future validation across independent datasets with varying preprocessing strategies.

Approximately 60% of features extracted from the optimal phase differed significantly from those extracted from MC reconstructions, regardless of whether mean or median averaging was used. An investigation into the dependence of features on volume and on other features demonstrated that unique features can be preserved depending on the 4D‐CT data evaluated. Only six features were common across reconstruction approaches following unsupervised feature selection, confirming the value of considering reconstruction techniques in radiomics analysis of 4D‐CT data.

Just two features differ between the MC_mean_ and MC_median_ reconstruction: *GLSZM Size Zone Non‐Uniformity Normalised (SZNUN)* and *GLSZM Small Area Emphasis*. These differences were consistent across all comparisons, suggesting that they are heavily affected by 4D‐CT image variability. *GLSZM SZNUN* assesses the evenness of zone size distribution and remains low when zone counts are equally distributed along zone sizes and the ROI is more homogenous. This feature has a low correlation with volume for all approaches and does not appear to be impacted by the ROI volume changes introduced in pre‐processing segmentation. *GLSZM SZNUN* remains only in the MC_median_ reconstruction and was correlated with *GLSZM Small Area Emphasis* for T_Op_ and MC_mean_. The median signal approach removes the tails of the distribution across the 4DCT phases, resulting in more homogenous regions.

Lafata et al. investigated the impact of spatial‐temporal resolution on radiomics features, identifying several features that showed an inverse dependence on SNR, that is, feature values tended to be higher in noisier images.^11^ In our study, we observed a similar pattern. While individual 4D phases have a high temporal resolution, mitigating the effects of motion, they typically suffer from a lower SNR compared to MC reconstructions, which contain roughly one‐third of the noise of individual scans^8^ One feature, *Run Length Non‐Uniformity*, known to increase with heterogeneity, was higher in T_Op_ (1032.85) than in MC_mean_ (948.73 and MC_median_ (936.29), suggesting that tumors on single 4DCT frames appear more heterogeneous than the same tumor on MC reconstruction due to increased noise. Other features highlighted by Lafata et al., *Entropy*, *Sum Entropy*, and *Short Run Emphasis*, did not show the same dependence in our study. However, features with a linear dependence on image SNR, *Id* and *Idm*, (analogous to *Homogeneity 1* and *Homogeneity 2* investigated in the spatial‐temporal resolution study), were increased for the MC reconstructions, confirming that increased noise inflates the apparent heterogeneity of individual phases, potentially misrepresenting true biological heterogeneity.

About 37.6% of features were volume‐dependent for the individual phase, compared to an average of 47.9% for the MC reconstructions. Among these, the MC_median_ reconstruction had the fewest remaining features after volume‐correlation filtering. As seen in Figure 4, the volume correlation coefficient for some features, particularly those from T_Op_ images, bordered the chosen cut‐off threshold, highlighting the sensitivity of feature inclusion to this choice. The statistical cut‐offs used were selected based on common practice but remain arbitrary and may not represent the ideal threshold for feature inclusion. Striking a balance is essential. Excluding volume‐correlated features helps avoid redundancy and prevents tumor size from dominating model predictions, but overly strict thresholds risk eliminating clinically relevant information. Two correlation thresholds were applied to optimize feature selection: a lower threshold (ρ ≥ 0.6) to remove moderately volume‐dependent features, and a higher threshold (ρ ≥ 0.8) to remove highly redundant features. While a singular cut‐off threshold value suffices for methodological comparisons, for clinical applications, it would be imperative to conduct comprehensive investigations into various cut‐off values to accurately comprehend their impact.

Additionally, we acknowledge that a formal sensitivity analysis was not performed to evaluate the impact of excluding tumors with volumes < 2 cm^3^. While this threshold was selected based on known limitations in radiomic analysis of small tumors, future work could explore how different volume exclusion criteria influence model performance and feature behaviour.

Each reconstruction retained a small set of features (11–14), with six features across all: First Order Kurtosis, GLCM Cluster Shade, GLCM Difference Variance, GLCM Inverse Variance, GLCM Maximal Correlation Coefficient (MCC), GLDM Large Dependence Low Gray Level Emphasis. The ability of these features to remain following the feature‐reduction process for all approaches demonstrates robustness to motion‐induced variability.

The T50 phase served as the reference phase for MC reconstructions, but T_Op_ did not always correspond to T50. In such cases, greater variability between features extracted from T_Op_ and the MC images would be expected. A potential improvement would be to generate the MC reconstructions using the patient‐specific T_Op_ as the reference. While we acknowledge this methodological inconsistency, it was not explicitly quantified, and the exact impact of reference phase selection on radiomic similarity and model performance from MC reconstructions remains unclear. Future work should include a dedicated comparison, reconstructing MC images using T_Op_ as the reference, to directly assess whether using patient‐specific phases for reconstruction influenced feature robustness.

While T_Op_ stability was assessed using a broad set of radiomic features, including both original and filtered images and both tumor and peri‐tumor regions, the radiomics analysis in this study included only original, unfiltered features extracted from the tumor region. This discrepancy might affect T_Op_ selection, but is unlikely to have a meaningful impact on our overall results. Future work could refine phase selection to match the exact feature set used in analysis.

The MC_Median_ model using Galileo DIR outperforms all other models. However, the improved performance can be explained due to differences at the volume correlation stage, where *GLCM Imc1* is removed for the T_Op_. For the MC_Median_ Galileo, *Imc1* is only selected 4 times more in cross‐validation than *GLDM Large Dependence Low Gray Level Emphasis (LDLGLE)*. Replacing Imc1 with *LDLGLE* in the multivariable Cox model so that the MC_Median_ signature is strongly correlated to that used in the optimal model results in decreased model performance (AIC = 268.01). This highlights the sensitivity of radiomics analysis.

### 4.2 Clinical implications

MC image reconstructions, particularly the median‐based approach, improve visual clarity of tumor boundaries and are less impacted by motion artefacts and the extremes of individual phases.^9^ This is highly beneficial in the clinic, reducing planning target volumes (PTVs) and improving observer variability.^17^ However, this advantage may come at the expense of valuable prognostic information. The pre‐processing threshold has a greater impact on the MC reconstructions, possibly explaining the higher feature exclusion rate at this stage due to reduced GTVs. Moreover, excluding pertitumoral information may limit the ability of radiomic models to capture infiltration characteristics, which have been shown to improve prognostic power.^12^, ^24^ Although the volume dependence of the features was accounted for, further work is required to investigate the impact of MC on features around the tumor.

In all models, prognostic features for distant failure were found, and radiomics models built using optimal single phase or MC_median_ images demonstrated improved performance over the clinical model according to AIC and CI. However, while these models showed significantly improved prognostic capability than the clinical model alone, the differences between the radiomics models were modest, with overlapping confidence intervals indicating no clear superiority of one approach over another.

Subtle differences in features within the radiomics signature are attributable to previously observed correlations. For Nifty MC_Median_, *First Order Maximum* correlates with *90 Percentile*, while *Long Run Emphasis* correlates with *Maximum Probability* for the T_Op_. Taking this into consideration, both the optimal and MC models appear to capture similar information; however, the optimal phase signature size is larger, and the MC models show significant predictors of distant failure. This is consistent across MC reconstruction approaches and despite algorithmic differences, all MC reconstruction approaches (NiftyReg, Elastix, and Galileo) preserved core information, albeit with varying signature sizes. These variations highlight the sensitivity of radiomic feature selection to different image reconstruction techniques.

A limitation of this study is the lack of an independent external validation cohort, which is essential for confirming the generalisability of radiomics‐based prognostic models. While external validation was not feasible within this study, we employed internal validation using a bootstrapping approach.^25^ Bootstrapping was applied consistently during both feature selection and model performance evaluation. The full dataset (*n* = 223) was used for unsupervised feature selection prior to the development of the supervised model. While this approach can raise concerns about potential information leakage, it is appropriate within the context of this study's methodological aim. The primary goal was to evaluate the impact of different motion correction strategies on radiomic model performance. For such comparative analyses, applying unsupervised feature selection to the full dataset, followed by internal validation, is methodologically consistent with a Type 1b analysis framework.^25^

It is also important to clarify that the prognostic utility assessed in this study serves as a comparative endpoint, rather than direct evidence of clinical applicability. While radiomics models outperformed the clinical model in terms of AIC and CI, this difference was subtle, and these results do not necessarily indicate that the identified signatures are ready for clinical translation. Future studies incorporating external validation are necessary to determine the true clinical utility of the radiomics‐based prognostic models for distant failure in NSCLC.

Finally, the study does not account for all factors influencing distant failure, notably, the interaction between radiomic features and radiation dose, which has shown predictive relevance in other work.^26^ Incorporating such variables in future studies will enhance clinical applicability.

Overall, only subtle differences were found between all signatures, in terms of radiomics features selected and prognostic performance. The optimal phase only slightly outperforms MC reconstructions, with temporal stability appearing to outweigh the cost of increased noise. The increased homogeneity of MC_median_ images might offer a benefit for the analysis of features that are sensitive to noise. When investigating subtleties in the MC reconstruction process, radiomic features are robust to changes in registration algorithm and differences within the feature selection process. However, MC_mean_ and MC_median_ approaches should not be used interchangeably, and the median approach offers more valuable information. Further, if MC is used as an alternative to phase radiomics, care must be taken due to the increased likelihood of volume correlation.

## 5 CONCLUSION

This study presents a comparative analysis of MC reconstructions and individual 4DCT phases, highlighting differences in radiomic feature values and their dependencies on tumor volume and other features. Our findings demonstrate that radiomic feature selection varies depending on the type of image used. Across different registration algorithms, MC‐based reconstructions produced radiomic models with comparable performance to those derived from a personalised 4DCT phase, with both approaches identifying features associated with distant failure. Both optimal phase and MC_median_ models showed a statistically significant improvement compared to clinical variables alone, regardless of the registration algorithm used to generate MC images. Motion compensation is a good alternative to a single 4DCT phase in radiomics, displaying robustness to the registration algorithm, with the median approach providing the most robust performance. While these results offer insights into how motion compensation affects radiomic feature extraction and model development, further studies are needed to assess their reproducibility and clinical relevance in independent datasets.

## AUTHOR CONTRIBUTIONS

Chelsea A. H. Sargeant: Formal analysis, Investigation, Methodology, Software, Writing – Original Draft Preparation, Writing – Review & Editing, Visualization. Angela Davey: Conceptualization, Data Acquisition, Methodology, Software, Supervision. Alan McWilliam: Conceptualization, Writing – Review & Editing, Supervision.

Drs. Alan McWilliam and Angela Davey are supported by Cancer Research UK via funding to the Cancer Research Manchester Centre [C147/A25254]. Dr. Alan McWilliam is also supported by Cancer Research UK RadNet Manchester [C1994/A28701] and the NIHR Manchester Biomedical Research Centre [NIHR203308].

## CONFLICT OF INTEREST STATEMENT

The authors declare no conflicts of interest.

## ETHICS STATEMENT

Our retrospective analysis of anonymized routine data was approved by institutional information governance and research ethics committee (REC references: 17/NW/0060 and 21/NW/0347).

## Supporting information

Supporting information

## Data Availability

The data analyzed in this study are subject to the following licenses/restrictions: ethical permission was not granted for general publication of the dataset. Requests to access these datasets should be directed to Dr. Alan McWilliam, alan.mcwilliam@manchester.ac.uk.
