# Supplementary material for: The suitability of radiomics extracted from 4DCT motion‐compensated reconstruction
Source: J Appl Clin Med Phys. 2025 Sep 29;26(10):e70254. doi: 10.1002/acm2.70254 (PMC12479209; doi:10.1002/acm2.70254)
Supplement: Supplementary file 1 — Supporting information [file ACM2-26-e70254-s001.pdf]

The suitability of radiomics extracted from 4DCT motion-compensated reconstruction.

Supplementary Material

1. Frequency of phase selected as optimal.

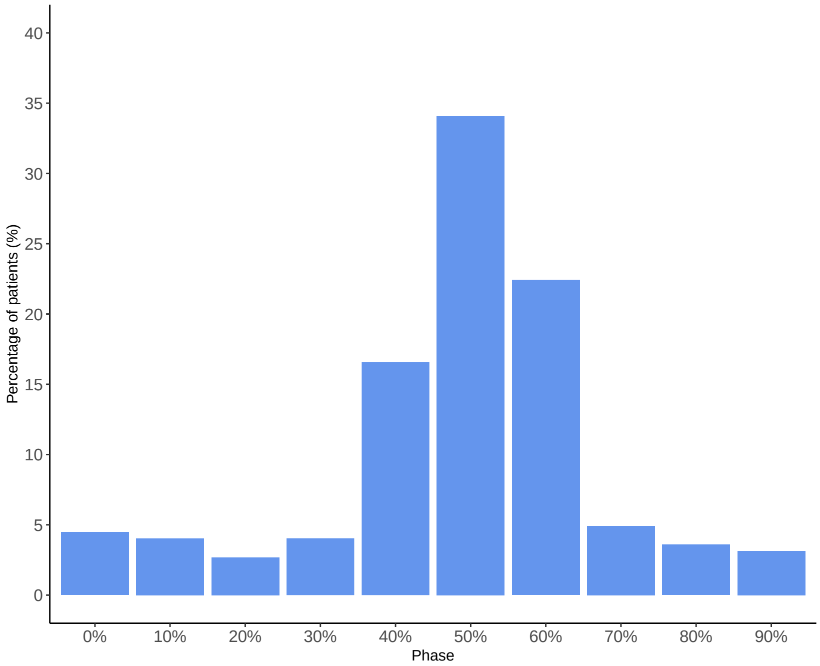

Figure 1. A histogram of the optimal phase selected for each patient. The phases close to exhale (40%-60%) were the optimal in most cases.

2. Changes in volume due to motion-compensated reconstruction

Table 1. The average absolute change in volume, in number of voxels, for each image type, optimal and motion compensated (MC) reconstruction.

	**Optimal**	**MC_{Mean} Nifty**	**MC_{Median} Nifty**	**MC_{Mean} Galileo**	**MC_{Median} Galileo**	**MC_{Mean} Elastix**	**MC_{Median} Elastix**
**Average absolute change in volume**	146.56	182.30	170.83	188.38	176.37	183.23	173.48

3. Differences in features extracted.

a. Analysis of variance results for Elastix and Galileo motion compensated reconstruction

Table 2. The number and percentage of features significantly differ when testing feature values between the optimal phase and Galileo and Elastix MC reconstructions. Like the Nifty MC, approximately 65% of features differ significantly from those extracted from the optimal phase and only 2.2% between the mean and median MC approach.

Feature Class	Optimal vs MC_{Mean} Galileo	Optimal vs MC_{Median} Galileo	MC_{Mean} vs MC_{Median} Galileo	Optimal vs MC_{Mean} Elastix	Optimal vs MC_{Median} Elastix	MC_{Mean} vs MC_{Median} Elastix
**First Order**	12 (66.7%)	11 (61.1%)	0 (0%)	12 (66.7%)	10 (61.1%)	0 (0%)
**GLCM**	17 (70.8%)	16 (66.7%)	0 (0%)	17 (70.8%)	17 (70.8%)	0 (0%)
**GLRLM**	11 (68.8%)	10 (62.5%)	0 (0%)	9 (56.3%)	10 (62.5%)	0 (0%)
**GLDM**	9 (64.3%)	9 (64.3%)	0 (0%)	9 (64.3%)	9 (64.3%)	0 (0%)
**GLSZM**	10 (62.5%)	10 (62.5%)	2 (12.5%)	10 (62.5%)	9 (56.3%)	2 (12.5%)
**NGTDM**	4 (80%)	4 (80%)	0 (0%)	4 (80%)	4 (80%)	0 (0%)
**Total**	**63 (67.7%)**	**60 (64.5%)**	**2 (2.2%)**	**61 (65.6%)**	**60 (64.5%)**	**2(2.2%)**

b. Second-order feature value differences

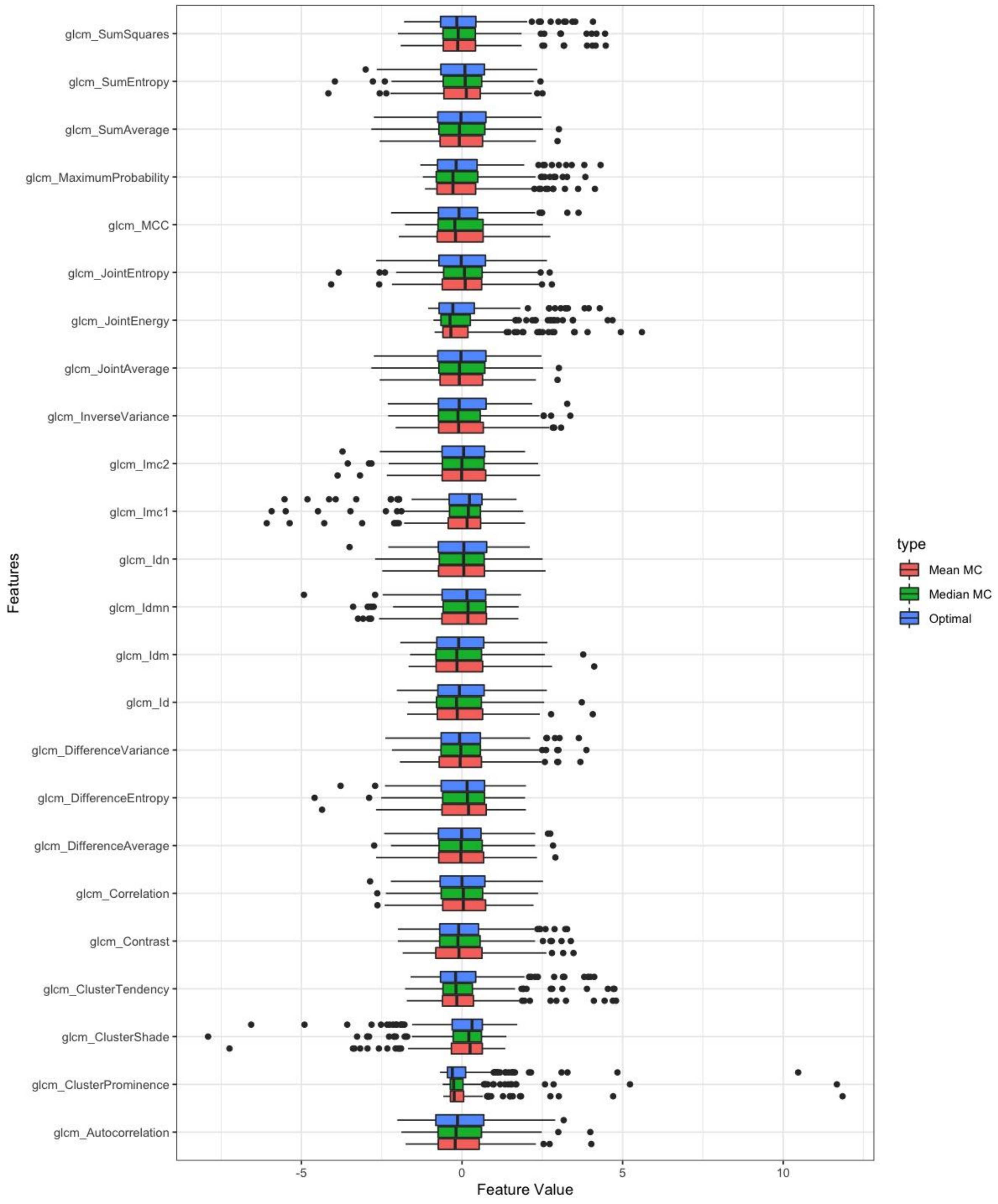

Figure 2. A comparison of symmetrical grey level co-occurrence matrix (GLCM) feature values extracted from the optimal phase, and the mean and median motion compensated (MC) reconstructions using NiftyReg.

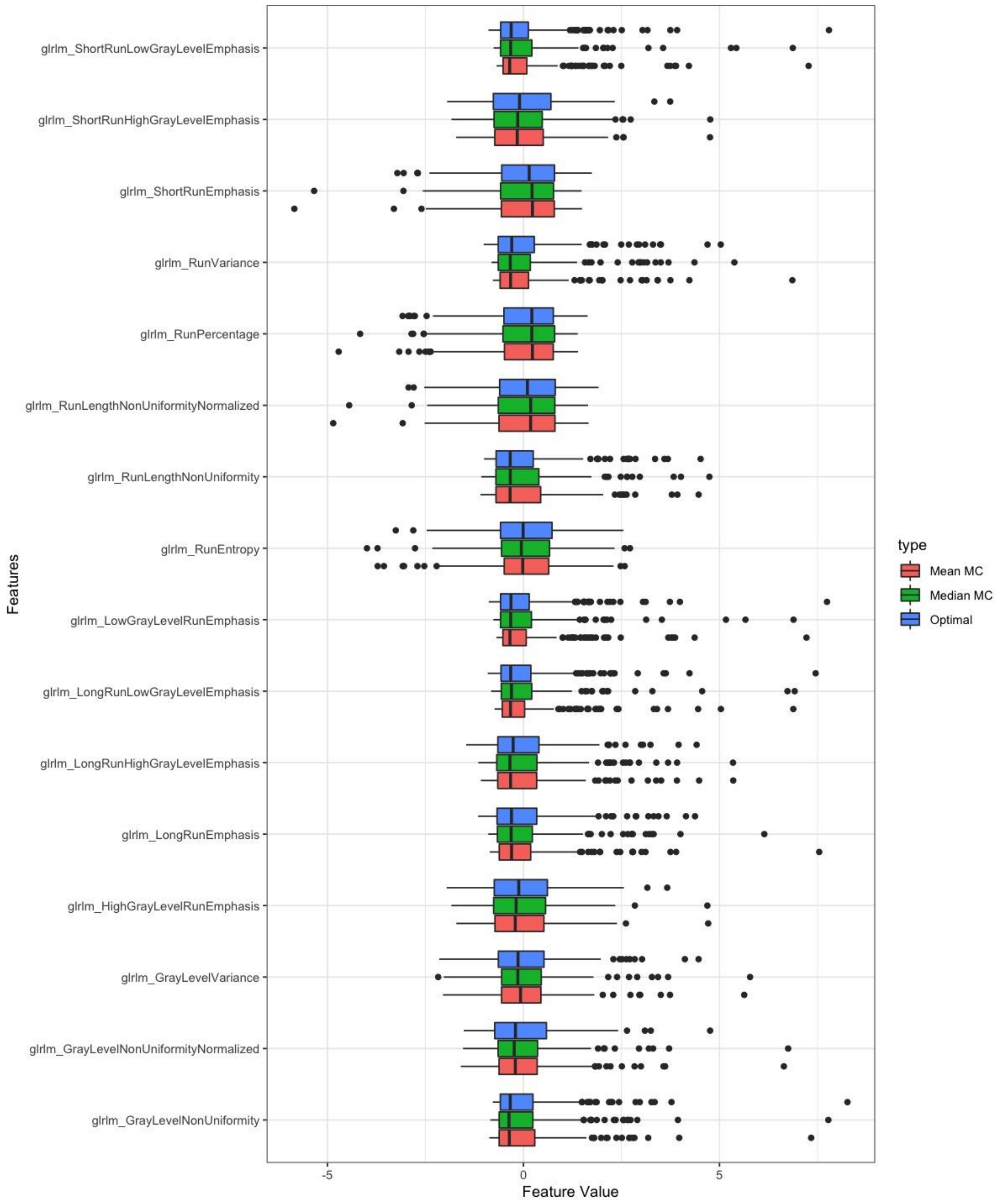

Figure 3. A comparison of grey level run length matrix (GLRLM) feature values extracted from the optimal phase, and the mean and median motion compensated (MC) reconstructions using NiftyReg.

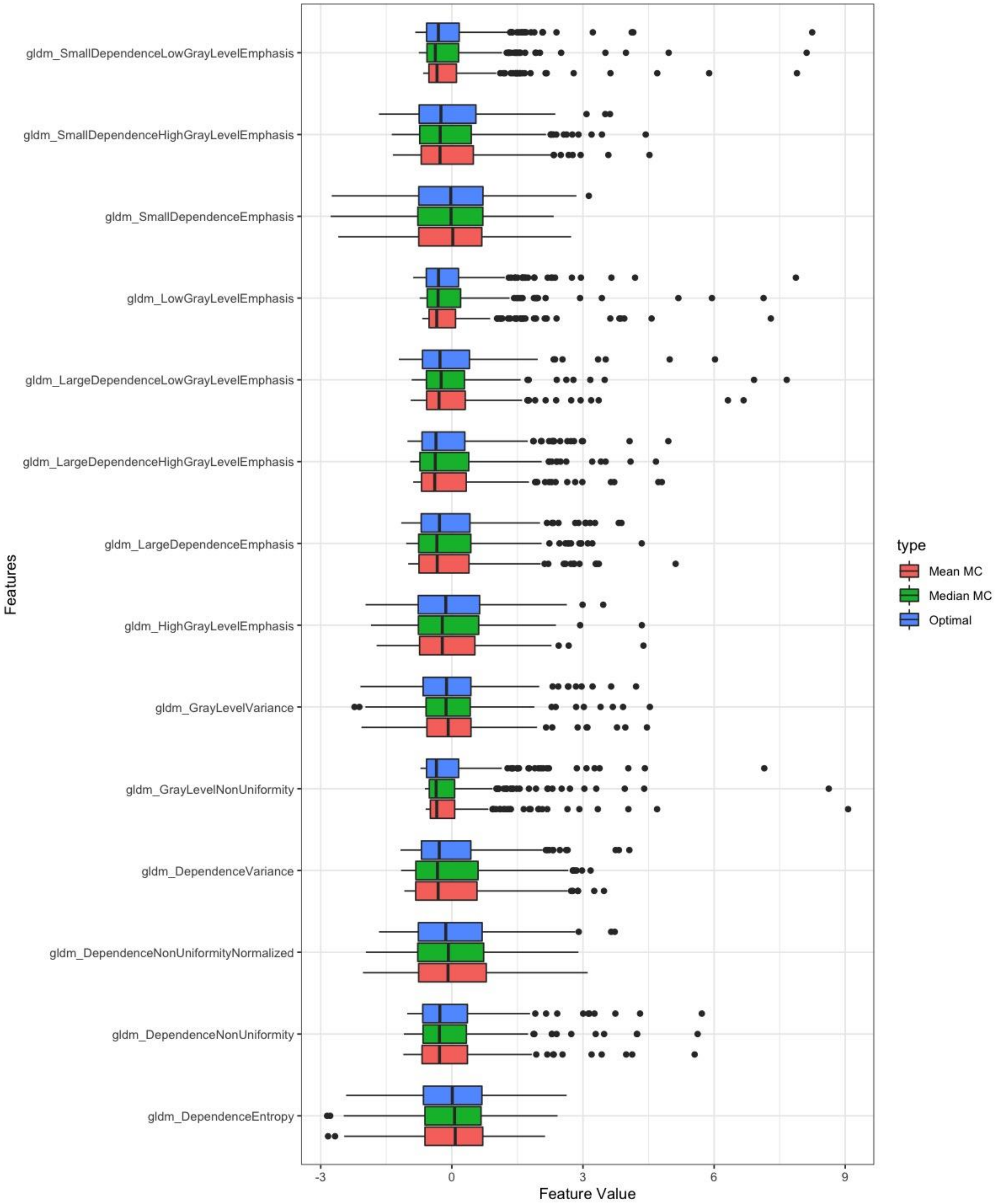

Figure 4. A comparison of grey level dependence matrix (GLDM) feature values extracted from the optimal phase, and the mean and median motion compensated (MC) reconstructions using NiftyReg.

23

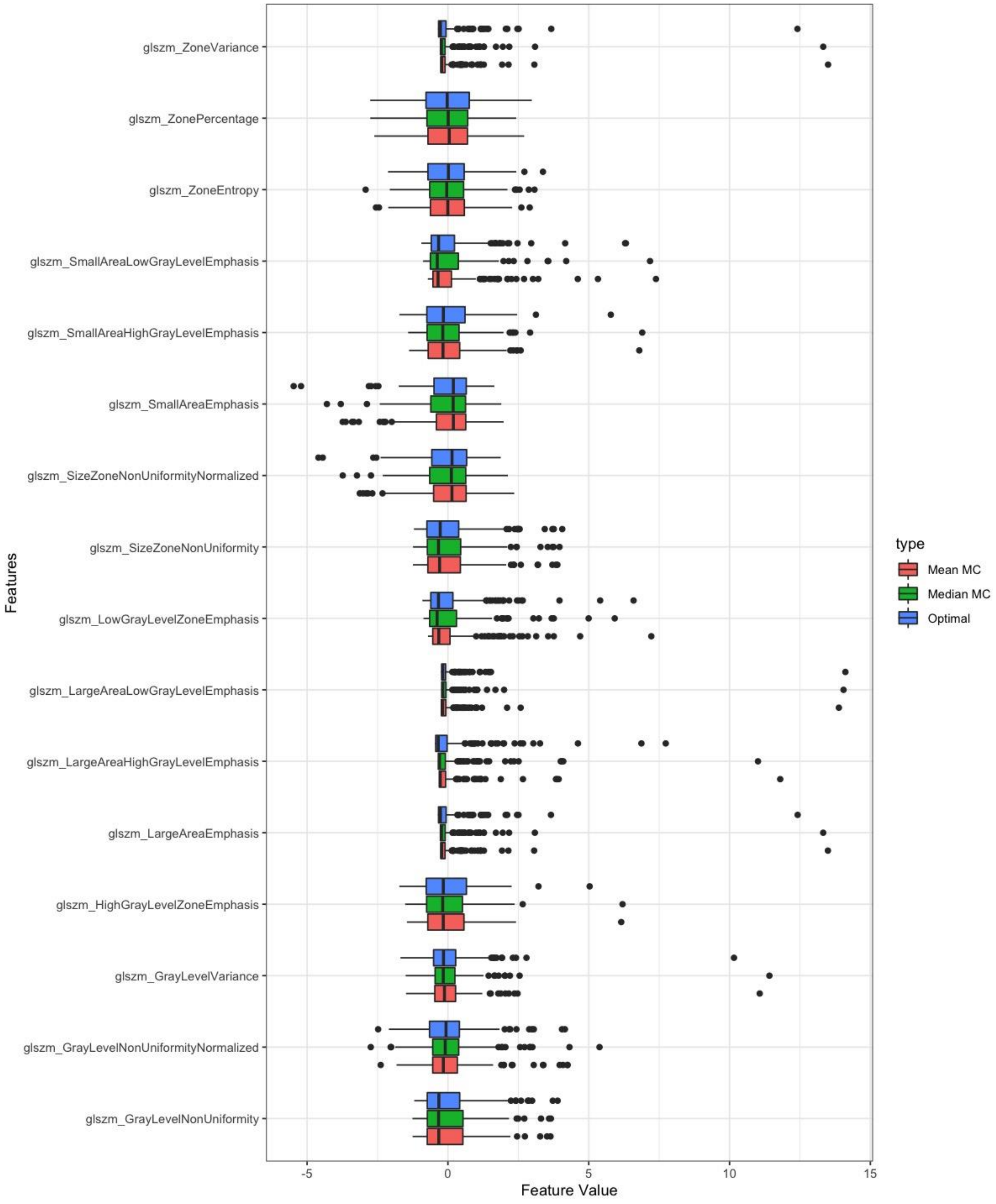

Figure 5. A comparison of symmetrical grey level size zone matrix (GLSZM) feature values extracted from the optimal phase, and the mean and median motion compensated (MC) reconstructions using NiftyReg.

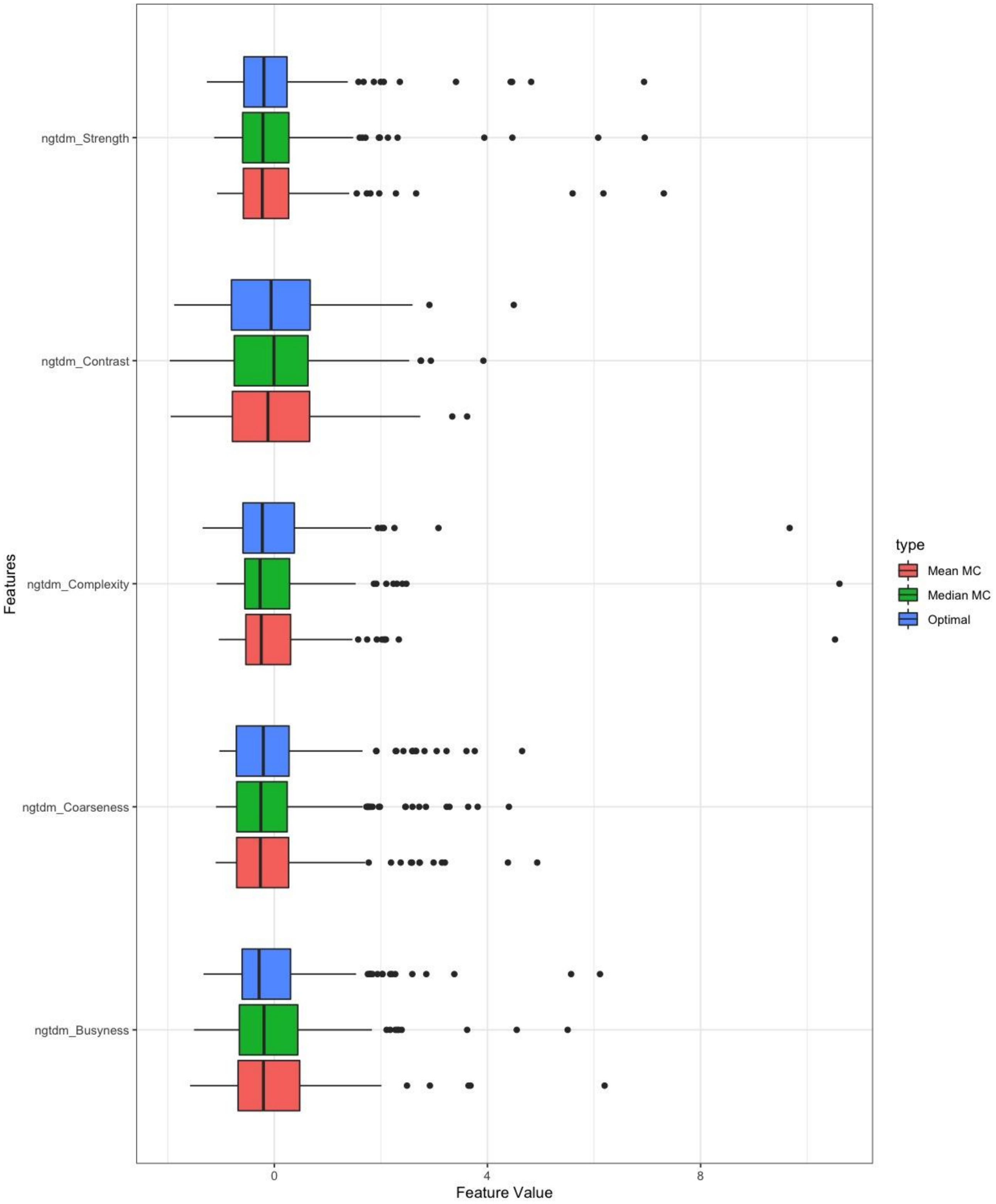

Figure 6. A comparison of neighbouring grey tone difference matrix (NGTDM) feature values extracted from the optimal phase, and the mean and median motion compensated (MC) reconstructions using NiftyReg.

4. Robustness to MC

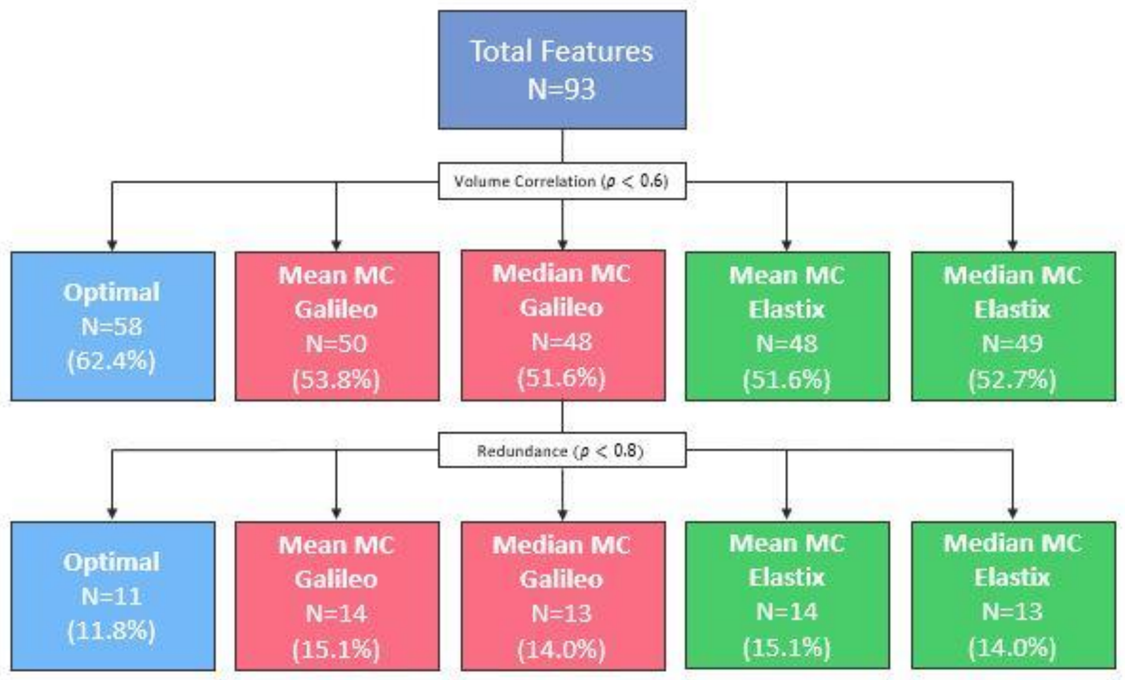

Figure 7. The number of features (N) remaining after each feature selection stage for optimal phase and mean and median MC reconstruction images generated using Galileo and Elastix deformable image registration. Similarly to NiftyReg MC, more features remain for the MC images than the single-phase image, despite the increased number of features correlated with volume.

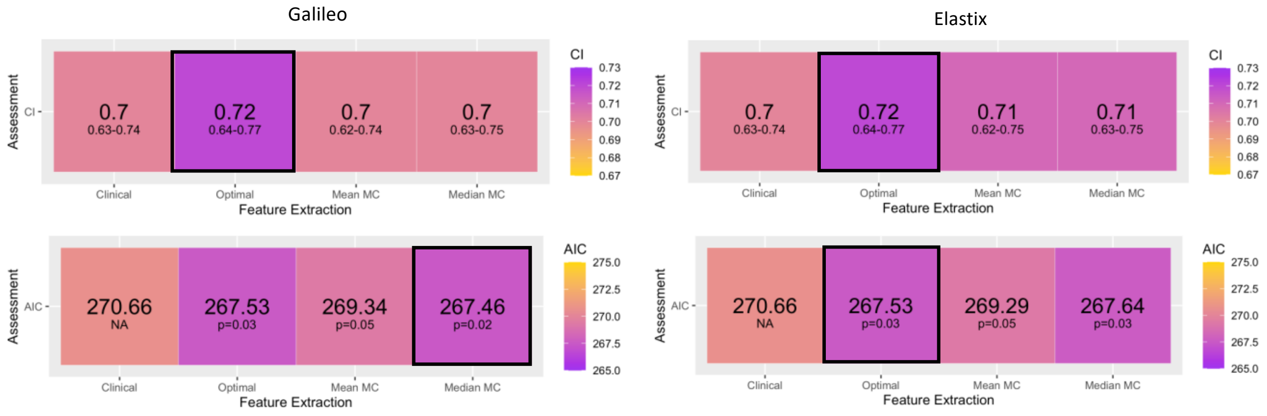

Figure 8. Median and 95% confidence interval of the concordance index (CI) across 500 bootstrap resamples (top), and the Akaike Information Criterion (AIC) for each model (bottom) for the Galileo MC (left) and Elastix MC (right). In both, the best-performing model is outlined by a black box. As shown in the main paper, the top-performing model used the optimal single phase when evaluated according to CI. However, the AIC results differ, in that the MCMedian model using Galileo registration is the top-performing model.

5. Clinical multivariable model without radiomic features

Table 3. Clinical multivariable model without radiomic features, clinical variables that are significant predictors of distant failure are highlighted in grey.

	HR (95% CI)	p-value
**log(Tumour volume)**	2.34 (1.31 - 4.20)	**0.004**
**Tumour motion amplitude**	0.44 (0.19 – 1.05)	0.063
**Lobe location (Lower reference)**	0.38 (0.18 – 0.84)	**0.017**
**Sex (Female reference)**	0.37 (0.17 – 0.84)	**0.017**
**Performance Status (0/1 reference)**		
**2**	1.40 (0.61 – 3.21)	0.429
**3**	2.45 (0.78 – 7.63)	0.124
**Age**	1.01 (0.97 – 1.06)	0.518
**T-stage (T1 reference)**	0.38 (0.16 – 0.90)	**0.028**
